# Prevalence of Obesity and Dental Caries in High School Adolescents during the First Decade of Saudi Vision 2030: A Cross-Sectional Study

**DOI:** 10.3390/children11050563

**Published:** 2024-05-08

**Authors:** Deema J. Farsi, Nada J. Farsi, Heba M. Elkhodary, Logain K. Alattas, Ali B. Alshaikh, Najat M. Farsi

**Affiliations:** 1 Department of Pediatric Dentistry, Faculty of Dentistry, King Abdulaziz University, Jeddah 21589, Saudi Arabia; hkhodary@kau.edu.sa (H.M.E.); nfarsi@kau.edu.sa (N.M.F.); 2 Department of Dental Public Health, Faculty of Dentistry, King Abdulaziz University, Jeddah 21589, Saudi Arabia; njfarsi@kau.edu.sa; 3 Department of Pedodontics and Oral Health, Faculty of Dental Medicine for Girls, Alazhar University, Cairo 11651, Egypt; 4 Department of Pediatric Dentistry, Tufts University, Boston, MA 02111, USA; logain.alattas@tufts.edu; 5 Saudi Board of Pediatric Dentistry, Riyadh Elm University, Riyadh 12611, Saudi Arabia; ali.alshaikh2022@student.riyadh.edu.sa

**Keywords:** dental caries, BMI, obesity, child, adolescent, Saudi Vision 2030

## Abstract

Saudi Vision 2030 was launched in 2016. Obesity and dental caries are both highly prevalent in Saudi adolescents and have been targeted by the Vision’s health initiatives. The aim is to assess their prevalence in adolescents during the first decade since the launch of the Vision. This cross-sectional study was conducted in Jeddah, Saudi Arabia using a stratified sample of 571 high school students, with an average age of 16.7 (0.6). Their height and weight were measured, and their body mass index (BMI) was calculated. The decayed, missed, and filled scores (DMFTs) were recorded after an oral examination. Non-parametric tests were used to assess the associations of DMFT with BMI, sex, and school type; and its predictors were assessed. One-third of males were overweight/obese compared with 22% of females. Males exhibited higher DMFTs than females. DMFTs were higher among public school students than among their private school counterparts. No significant association was observed between DMFT and BMI. Sex and school type were significant predictors of DMFT. The prevalence of obesity has slowly decreased in adolescents, but the prevalence of dental caries has not. There was no significant relationship between these conditions. Saudi Vision 2030’s current preventive/educational initiatives may be more effective in combating obesity than dental caries.

## 1. Introduction

Obesity and dental caries are highly prevalent health problems in children worldwide [1,2]. According to the World Health Organization, more than 500 children have dental caries and 160 million live with obesity [3,4]. Both conditions are chronic with deleterious impacts on health. These diseases are multifactorial and share common dietary, socioeconomic, genetic, and environmental risk factors [5]. Furthermore, family tradition and lifestyle have been associated with both conditions [6]. In Saudi Arabia, the prevalence of obesity and dental caries has rapidly increased in recent years. The prevalence of obesity in adolescents is estimated to be 20.2%, reaching 34.7% when combined with overweight [7]. Dental caries is also highly prevalent in Saudi children. According to the Saudi Ministry of Health, 93.7% of 12-year-old children have dental caries [8]. The national prevalence of caries in adolescents is estimated to be 72% [9].

Diet is a factor that contributes to both childhood obesity and dental caries [5]. The consumption of high-sugar diets has been linked to the development of several childhood conditions, e.g., type II diabetes, obesity, and dental caries. High-sugar diets, especially those fostered with sweetened carbonated drinks, are commonly consumed in adolescence and have been linked to obesity and dental caries [10,11,12]. Moreover, it has been recently suggested that obesity influences oral microflora and salivary composition, and, thus, may promote dental caries [13]. On the basis of this, several studies have investigated the relationship between both conditions in different age groups, but the results have been inconclusive and controversial [6,10,14,15].

Saudi Vision 2030 is Saudia Arabia’s strategic framework with the primary objective of reducing its dependence on oil and diversifying its economy. It is regarded as one of the most influential regional reforms. The first details were shared in 2016, after an announcement was made by Saudi Crown Prince Mohammed Bin Salman [16]. One of the Vision’s objectives is healthcare promotion, in which obesity is identified as a health risk factor [17]. Several initiatives have been introduced to reduce obesity, including the establishment of nutrition clinics, the RASHAKA program, and public and in-school initiatives related to physical activity like Sports For All [17,18,19,20]. Besides obesity, dental caries has received increasing attention from the Saudi Ministry of Health recently. A large initiative called “Prevent Tooth Caries” has served over 1 million students in more than 5,300 schools by 2018 [21]. The National Transformation Program, one of Vision 2030’s Vision Realization Programs, includes an initiative to invest in dental clinics to serve local communities [17].

Obesity and dental caries have been investigated in Saudi children. This study was conducted to assess their prevalence in high school adolescents in Jeddah, Saudi Arabia in the first decade after the launch of Saudi Vision 2030. A comparison with findings from previous studies could indirectly assess the effectiveness of the health initiatives of the Vision Realization Programs and aid in supporting programs targeting both conditions in this critical population on the verge of adulthood.

## 2. Materials and Methods

### 2.1. Ethics Statements

The Ethics Committee of the Faculty of Dentistry at King Abdulaziz University (KAUFD) approved this study (approval number: 064-06-20). Informed consent was obtained from the parents of all participants.

### 2.2. Study Design

This cross-sectional study targeted high school adolescents. It was part of a three-arm project, where the prevalence of obesity and dental caries was investigated among school students in Jeddah. The study was conducted between September 2022 and March 2023.

### 2.3. Subjects

Eleventh graders were chosen to represent a population of 44,016 Jeddah high school students with permanent dentition. Males comprised 51.1% of the study participants. Students were enrolled in 410 high schools, 57.8% of which were public schools. As schools in Jeddah are segregated by sex and geographic location, a list of male and female schools for each city section was created. At least one public school and one private school were randomly selected from each list. A total of 20 schools were visited (12 public and 8 private schools to maintain a 60:40 public-to-private school ratio) after obtaining permission from the Ministry of Education in Jeddah and the principals of the selected schools.

Each school was visited twice. During the first visit, a parental consent form with a brief description of the study was distributed to all eleventh graders. The form also included questions to collect demographic data. Anonymity was assured, and participation was voluntary. During the second visit, an examination was performed on eligible students. The inclusion criteria were healthy students, aged between 15 and 18 years, and those who brought back a signed consent form with the answered demographic questions. Exclusion criteria were students with special healthcare needs, younger than 15 or older than 18 years, and students who did not have a consent form signed by their parents or who refused to participate in the study. A total of 571 students were enrolled in the study.

### 2.4. Anthropometric Measurements

A single nonelastic measuring tape and an electronic weight scale were used for all participants. For weight measurement, students were instructed to remove their shoes, jackets, and accessories. Waist circumference (WC) was measured while the students were standing at the end of normal expiration; the reference point was the highest point on the iliac crest. To measure height, the students stood barefoot and looked straight ahead. The highest point on the head was marked on a white cardboard fixed against the wall. Tape was used to measure the distance between the marked point and the floor. Two readings were obtained for each measurement and averaged for further analysis. The body mass index (BMI) of each student was calculated as the ratio of weight to height squared (kg/m^2^). The waist-to-height ratio (WHtR) was calculated by dividing the WC (cm) by height (cm).

All anthropometric measurements were taken by two investigators after calibration. For the intra-examiner reliability assessment, 10 patients from KAUFD were examined twice by each investigator at 10-day intervals (kappa score, 0.86). For the inter-examiner reliability assessment, 15 patients from KAUFD were examined by both investigators (kappa score, 0.83). In case of any disagreement, a consensus was reached through repeated measurements.

### 2.5. Dental Examination

Two calibrated investigators performed oral examinations. Calibration was based on a detailed rubric for caries detection according to the World Health Organization criteria, where the decayed, missed, and filled scores (DMFTs) for the permanent teeth were recorded [22]. For the intra-examiner reliability assessment, 15 patients from KAUFD were examined twice with a 10-day interval (kappa score, 0.90). For the inter-examiner reliability assessment, the first readings of 15 patients were evaluated (kappa score, 0.88). In cases of disagreement, a consensus was reached by repeating the measurements.

Disposable instruments were used for the examination: a sterile, flat-surface intraoral mirror, a round-ended dental probe, gauze, and cotton rolls. Teeth with enamel surface structural losses and/or restorations exhibiting secondary caries around the margins were marked as decayed. Teeth with temporary restorations were marked as decayed. However, teeth with sealants and white spot lesions were marked as sound. A confidential report explaining the students’ oral health status and treatment needs was sent with a referral letter to KAUFD.

### 2.6. Statistical Analysis

Approximately 40% of the adolescents in Saudi Arabia are overweight or obese [14]. Therefore, the following criteria were used to estimate the appropriate sample size:

– The power of the study was set at 85%, and the alpha was set at 5%.
– The percentage frequency of the outcome factor was set at 30% with a 3% confidence limit.

According to these calculations, the estimated sample size was 428 students, divided equally between males and females.

Descriptive statistics were used to summarize the sociodemographic and biometric characteristics of participants. The proportion of obese participants was estimated and stratified by sex to assess their association.

Children were classified according to age- and sex-specific BMI percentiles as follows: underweight (BMI below the 5th percentile), normal weight (BMI within the 5th–84th percentiles), overweight (BMI within the 85th–94th percentiles), and obese (BMI at or above the 95th percentile) using the Saudi BMI percentiles and standard cut-off points [23,24]. Additionally, the students were classified as obese or non-obese based on WC. Obese children were those with WC at or above the 90th percentile for age and sex, according to the percentiles and cut-off points suggested by Fernandez et al. and Bassali et al. [25,26]. A WHtR cut-off point of 0.5 was used to define abdominal obesity [27].

The normality of the DMFT was assessed using a histogram, Skewness and Kurtosis test, and a normal quantile–quantile plot, all of which indicated the non-normality of the DMFT. Therefore, non-parametric tests were used.

The prevalence of BMI was stratified by sex and school type, and the associations between BMI and each of these two variables were assessed using the chi-square test. Furthermore, the distribution of DMFTs according to sex, school type, and BMI was assessed. The mean DMFTs among each variable’s categories were assessed using the two-sample *t*-test.

A zero-inflated negative binomial regression model was employed to determine the predictors of dental caries among the participants. The analysis was conducted in two steps: univariate regression analysis to explore the relationship between each predictor and the outcome variable and multivariate regression analysis to adjust for potential confounding factors.

Statistical significance was set at a *p*-value of ≤0.05. The data for the statistical tests were processed using Stata software (version 12.1; StataCorp LP, College Station, TX, USA).

## 3. Results

As shown in Table 1, the sample consisted of 571 children with a mean age of 16.7 ± 0.6 years, of whom 53.1% were male. More than half the participants attended public schools (56.6%). The household monthly income levels were mostly within the range of SAR 4000–38,000 (87.0%). The prevalence of obesity according to BMI was distributed as follows: underweight, 7.0%; normal weight, 65.2%; overweight, 12.6%; and obese, 15.2%. Moreover, WC was used to classify participants as non-obese (89.1%) and obese (10.9%), in addition to the WHtR (non-obese, 89.1%; obese, 10.9%).

Table 2 presents BMI distribution, stratified by sex and school type. BMI was associated with sex; 67% of males were in the underweight/normal BMI category compared with 78% of females. Approximately 33% of males were classified as overweight/obese compared with 22% of females. However, school type was not associated with BMI.

The distribution of DMFTs stratified by sex, school type, and BMI categories is presented in Table 3. The mean DMFT was higher in males than in females (4.9 ± 4.1 versus vs. 4.1 ± 3.3; *p* = 0.049). The DMFT was also higher among public school students than among private school students (5.1 ± 3.9 vs. 3.7 ± 3.5, *p* < 0.001). There was no association between DMFT and BMI (*p* = 0.156).

Table 4 illustrates the predictors of DMFT among the participants. Sex demonstrated an association with DMFT, with females showing a lower DMFT than males, as indicated by a coefficient of −0.25 (95% confidence interval [CI]: −0.4, −0.1) in both univariate and multivariate models. The type of school attended was also a predictor of DMFT. Students from private schools had a lower rate of dental caries than their public school counterparts, with a multivariate coefficient of −0.16 (95% CI: −0.3, −0.04). Nationality, health status, parental education, income level, and BMI were not associated with DMFT.

## 4. Discussion

This cross-sectional study assessed the prevalence and distribution of obesity in adolescents in Jeddah. It also assessed the prevalence of dental caries in permanent dentition and studied the relationship between obesity and dental caries. The mean BMI was 23.7 ± 6.3, with most adolescents in the normal weight category. The prevalence of obesity was higher in males than in females. Similarly, caries activity was higher in males than in females, in addition to adolescents attending public schools. The mean DMFT was 4.5 ± 3.8. Approximately 20% of the participants had a DMFT of 0, and four participants had a score of ≥15. No association was observed between obesity and caries activity.

Obesity is linked to several health problems including cardiovascular diseases, sleep apnea, and cancer [28]. Internationally, obesity is highly prevalent among adolescents: 14.06% in China and 21.5% in the United States [29,30]. There are various means to measure obesity, one of which is BMI. On the basis of BMI, 27.8% of the adolescents in the current study were overweight/obese. This is lower than that found in a similar study conducted in Jeddah eight years earlier (40%) [14]. This percentage is also lower than the findings of two other local studies conducted in the Eastern Province (35.3%) and Riyadh (34.7%) [7,31]. However, more recent studies have documented a lower obesity prevalence than that in the current study [32,33]. BMI is the most commonly used obesity measure in studies on Saudi children. Although a useful tool, BMI has drawbacks, as it cannot quantify fat directly and does not differentiate between lean and fatty masses. Visceral fat, which reflects harmful internal fat, can be better estimated by measuring WC [34]. According to WC, 10.9% of adolescents were obese, which is lower than that previously assessed [14,35]. Another non-invasive obesity measure that has gained attention lately is the WHtR, which is used to estimate central adiposity and predict the risk of cardiometabolic diseases [36]. The prevalence of obesity was 10.9% according to the WHtR, which is lower than that reported in previous studies conducted in Riyadh and Jeddah [35,37].

The prevalence of obesity is higher in males than in females. This result is consistent with the findings of previous studies [14,31,32,37]. Focus on body image increases in adolescence owing to active cognitive and physical changes [38]. Females tend to be more concerned with body image and are more prone to compare themselves with others than males [39]. This concern may lead them to eat less, eat healthier, and/or engage in fat-burning activities, e.g., walking and swimming. Moreover, increased testosterone levels in males during adolescence lead to denser bones and bulkier muscle mass, which may lead to increased weight and, thus, a higher BMI [40]. These are plausible explanations for the lower prevalence of obesity among females. In terms of school type, there was no difference between public and private schools in this study. In previous studies, children from private schools and high-income families were found to have higher rates of obesity than their counterparts [7,14]. An explanation for the current lack of difference may be attributed to the increased emphasis on physical activity promoted and made publicly available, and recent fitness initiatives that are inclusive of all social classes. Furthermore, the Ministries of Health and Education have implemented several physical activity programs in both school types, and school canteens have been encouraged to provide healthier food options.

Dental caries is the most prevalent childhood disease and is one of the most unmet health needs [3,41]. It is highly prevalent among Saudi children, where 72% of adolescents were estimated to be affected compared with 53.8% worldwide [9,42]. In the current study, approximately 80% of participants had a DMFT of ≥1. Moreover, the mean DMFT in the current population was 4.5 (3.8), which is higher than that in studies published locally a decade ago [14,43]. Regarding distribution, caries activity was significantly higher in males than in females, similar to a recent study that identified behaviors associated with poorer oral health among males [44]. In contrast, previous studies found females to have significantly higher caries activity than males [14,45]. Concerning school type, adolescents in public schools had significantly higher caries experience than did those in private schools. This result is consistent with the findings of previous studies [14,15].

The predictors of dental caries were also investigated. Nationality, health status, parental education, income, and BMI were not predictors of increased caries activity. However, male sex and studying at a public school were predictors of a higher DMFT. As dietary habits and oral hygiene practices were not investigated, it was difficult to ascertain the cause of the sex-based discrepancies. However, one could speculate about the attention females pay to their looks and self-image at this age, which is usually higher than that of males [39]. Additionally, teenage girls have been found to engage in better oral hygiene practices than their male counterparts [46]. Although family income did not have a direct influence on caries activity, affluence often had an inverse relationship with dental caries [1]. The difference in DMFT between school types may be attributed to earlier and better access to preventive measures and resources among the more affluent group [47].

As obesity and dental caries share some common risk factors, the relationship between them seems logical. The consumption of high-sugar diets, processed foods, and carbonated sweetened soft drinks, which is common in adolescence, places adolescents at a greater risk of gaining weight and developing dental caries [11,12,48]. Recent local studies found a statistically significant association between BMI and dental caries in Saudi adolescents [49,50]. An association was also found in international studies [51,52]. However, in the current study, there was no association between both conditions. This may be attributed to the fact that both conditions are multifactorial in nature and their association is more complex than can be attributed to a single common dietary risk factor. Even with a poor diet as a risk factor, if it is high in saturated/trans-saturated fat and not sugar, it may increase the likelihood of obesity, but not dental caries. Similar to the current findings, previous studies have found no relationship between the two conditions [14,53,54].

Adolescence is commonly linked to problematic behaviors and pronounced uncertainty. However, this important period encompasses biological, psychological, and social growth. Furthermore, it is a transitional period into adulthood and a good time to lay the foundation for good health. Obesity and dental caries in adolescents are likely to have health consequences that persist into adulthood. Thus, controlling them at a young age will not only have short-term benefits but also increase the likelihood of a healthier adulthood. It is estimated that 15.8% of the Saudi population is between 10 and 19 years of age [55]. This study’s findings are promising in terms of obesity. The obesity rate has declined slightly since the previous local publications. Although fast-food chains continue to grow in Saudi Arabia, healthy food options and chains are rapidly growing in popularity. With Saudi Vision 2030, health awareness among adolescents has increased, especially through campaigns capitalizing on social media. Physical activity initiatives have enhanced the lives of many youths, and sports have become more popular than before. It is logical to predict that the decrease in obesity prevalence will continue with the attention given to health in the country. However, these findings were not positive for dental caries. Caries prevalence in Saudi adolescents remains high, especially among public school students. Unlike obesity, dental caries is more challenging to reverse. Current public health campaigns targeting schoolchildren may target populations beyond the point of feasible change. Thus, a shift toward more effective and earlier prevention is warranted. This can be achieved by targeting pregnant women and parents of young children, such as those attending well-baby clinics or early childhood care. The benefits of promoting oral health in the nondental workforce should not be underestimated. Training primary care providers in oral health (examination and prevention) can substantially enhance children’s oral health. Once time-sensitive prevention programs are implemented, current oral health initiatives can foster changes in oral health. Finally, campaigns tailored specifically for adolescents, particularly in the presence of social media, to warn them about the harmful effects of high-sugar diets, especially those who frequently consume soft drinks, can aid in controlling both obesity and dental caries.

The current study had some limitations. The cross-sectional design demonstrated findings only at the time of examination. It would have been ideal if participants had been followed up over time to assess consecutive changes in obesity and dental caries levels. Second, dietary habits, physical activity, and the use of Vision initiatives were not investigated. They would have provided better insight into the association with causative factors; however, this was beyond the scope of the current study. Nevertheless, this study had several strengths. First, the methodology was meticulous and thorough. Second, to compensate for the limitations of BMI, more than one measure was used to assess the prevalence of obesity. Third, the study focused on discussing findings from local studies to accurately relate them to the current findings. Finally, the study was comprehensive and included children of both sexes, attending both school types, and recruited from all districts in the city.

## 5. Conclusions

There has been a slight decrease in the prevalence of obesity among Saudi adolescents since the launch of Saudi Vision 2030 in 2016. However, the incidence of dental caries remains high in this population. Current health initiatives targeting dental caries may be less efficient or underused. It is crucial that additional oral health prevention programs be introduced, and their effectiveness assessed. Further longitudinal studies are required to assess the long-term effects of the Vision’s health initiatives. Furthermore, it is suggested that the causative factors for both conditions be investigated to better tailor future interventions.

## Figures and Tables

**Table 1:** Sociodemographic and biometric characteristics of the participants.

Variable	N (%) * N = 571
Age, mean (SD)	16.7 (0.6)
Sex	
Male	303 (53.1)
Female	268 (46.9)
Nationality	
Saudi	515 (90.2)
Non-Saudi	56 (9.8)
School type	
Public	323 (56.6)
Private	248 (43.4)
Health problems	
No	96 (83.2)
Yes	475 (16.8)
Father education	
High school or less	89 (15.6)
University	363 (63.6)
Postgraduate	119 (20.8)
Mother education	
High school or less	115 (27.2)
University	365 (63.9)
Postgraduate	51 (8.9)
Income (SAR)	
<4000	40 (7.0)
4000–38,000	497 (87.0)
>38,000	34 (6.0)
DMFT, mean (SD)	4.5 (3.8)
Anthropometric Measurements	
Height (cm), mean (SD)	164.5 (9.1)
Weight (Kg), mean (SD)	64.5 (19.2)
WC (cm), mean (SD)	86.4 (15.2)
BMI, mean (SD)	23.7 (6.3)
WHtR, mean (SD)	0.5 (0.1)
Obesity prevalence	
BMI	
Underweight (0–4.9)	40 (7.0)
Normal (5–84)	372 (65.2)
Overweight (84–94)	72 (12.6)
Obese (95 and above)	87 (15.2)
WC	
Non-obese (0–89.9)	509 (89.1)
Obese (90 and above)	62 (10.9)
WHtR	
Non-obese (<0.55)	509 (89.1)
Obese (0.55 above)	62 (10.9)

DMFT: decayed, missed, filled teeth index; BMI: body mass index; WC: waist circumference; WHtR: waist–height ratio. SAR 1 = USD 0.27. * Number (percentage), unless indicated otherwise.

**Table 2:** Prevalence of BMI obesity stratified by sex and school type.

Variable	Sex		*p*-Value ^	School Type		*p*-Value ^
	Male	Female		Public	Private	
BMI classification						
Underweight/Normal	203 (67.0)	209 (78.0)	0.003	238 (73.7)	174 (70.2)	0.352
Overweight/obese	100 (33.0)	59 (22.0)		85 (26.3)	74 (29.8)	

^ Chi-square test.

**Table 3:** Distribution of DMFT by sex, school type, and BMI.

Variable	DMFT		*p*-Value *
	Mean (SD)	Median (IQR)	
Sex			
Male	4.9 (4.1)	4 (1, 8)	0.049
Female	4.1 (3.3)	4 (1, 6)	
School type			
Public	5.1 (3.9)	5 (2, 8)	<0.001
Private	3.7 (3.5)	4 (0, 6)	
BMI			
Underweight/Normal	4.6 (3.8)	4 (1, 7)	0.103
Overweight/obese	3.9 (3.5)	4 (0, 7)	

* Wilcoxon Rank-Sum Test.

**Table 4:** Predictors of dental caries (DMFT) among the participants (zero-inflated negative binomial model) -best fit-.

Variable	N	Univariate Regression Coefficient (95 CI% *)	Multivariate Regression Coefficient (95 CI% *)
Sex			
Male	303	1.0	1.0
Female	268	−0.25 (−0.4, −0.1)	−0.25 (−0.4, −0.1)
School type			
Public	323	1.0	1.0
Private	248	−0.19 (−0.3, −0.1)	−0.16 (−0.3, −0.04)
Nationality			
Saudi	515	1.0	1.0
Non-Saudi	56	−0.13 (−0.3, 0.1)	−0.05 (−0.2, 0.1)
Health			
No	96	1.0	1.0
Yes	475	0.06 (−0.1, 0.2)	0.08 (−0.1, 0.2)
Father education			
High school or less	89	1.0	1.0
University	363	0.15 (0.0, 0.3)	0.00 (−0.2, 0.2)
Postgraduate	119	0.01 (−0.2, 0.2)	−0.06 (−0.3, 0.1)
Mother education			
High school or less	115	1.0	1.0
University	365	0.05 (−0.1, 0.2)	−0.06 (−0.2, 0.1)
Postgraduate	51	−0.02 (−0.2, 0.2)	0.04 (−0.2, 0.3)
Income (SAR)			
<4000	40	1.0	1.0
4000–3800	497	0.07 (−0.1, 0.3)	−0.01 (−0.2, 0.2)
3800	34	−0.23 (−0.5, 0.1)	−0.15 (−0.5, 0.2)
BMI			
Underweight/Normal	412	1.0	---
Overweight/obese	159	−0.002 (−0.1, 0.1)	
BMI			
Normal	372	1.0	1.0
Underweight	40	−0.07 (−0.3, 0.1)	−0.04 (−0.2, 0.2)
Overweight/obese	159	−0.01 (−0.1, 0.1)	−0.01 (−0.1, 0.1)

* 95% CI: 95% Confidence Interval.

## Data Availability

All data generated for this study belong to the current research team and are available from the corresponding author upon reasonable request to protect the data privacy and restrict unauthorized use.
